# Contributions of lignification, tissue arrangement patterns, and cross-sectional area to whole-stem mechanical properties in *Arabidopsis thaliana*

**DOI:** 10.1007/s10265-024-01543-2

**Published:** 2024-04-26

**Authors:** Mariko Asaoka, Eric Badel, Ali Ferjani, Kazuhiko Nishitani, Olivier Hamant

**Affiliations:** 1 Laboratoire de Reproduction Et Développement Des Plantes, Université de Lyon, UCB Lyon 1, ENS de Lyon, INRAE, CNRS, 46 Allée d’Italie, 69364 Lyon Cedex 07, France; 2 Department of Biological Sciences, Faculty of Science, Kanagawa University, Yokohama-Shi, Kanagawa, 221-8686 Japan; 3 Department of Biology, Tokyo Gakugei University, Koganei-Shi, Tokyo, 184-8501 Japan; 4 Université Clermont Auvergne, INRAE, PIAF, 63000 Clermont–Ferrand, France

**Keywords:** *Arabidopsis thaliana*, Bending test, Inflorescence stem, Lignification, Vascular bundle

## Abstract

Plant cells withstand mechanical stress originating from turgor pressure by robustly maintaining the mechanical properties of the cell wall. This applies at the organ scale as well; many plant stems act as pressurized cylinders, where the epidermis is under tension and inner tissues are under compression. The *clavata3 de-etiolated3* (*clv3-8 det3-1*) double mutant of *Arabidopsis thaliana* displays cracks in its stems because of a conflict between the mechanical properties of the weak epidermis and over-proliferation of inner stem tissues. In this work, we conducted three-point bending tests on various *Arabidopsis thaliana* mutants, including those displaying the stem cracking phenotype, to examine the differences in their mechanical properties. The *clv3-8 det3-1* double mutant exhibited reduced stem stiffness, consistent with reduced differentiation due to the *clv3-8* mutation. Yet, in *clv3-8*, stem cross-sectional area was increased associating with the increase in vascular bundle number, and stem cross-sections displayed various shapes. To uncouple the contribution of geometry and cell-wall differentiation to the mechanical properties of the whole stems, we tested the contribution of lignified fibers to stem stiffness. In order to suppress lignin deposition in stems genetically, we generated multiple higher-order mutants by crossing *clv3-8* and/or *det3-1* with *nst1-1 nst3-1,* in which lignin deposition is suppressed. Stem stiffness was reduced markedly in all *nst1-1 nst3-1* mutant backgrounds. Overall, our results suggest that stem stiffness relies on the presence of differentiated, lignified, fiber tissue as well as on the alignment or spatial distribution of vascular bundles within the stem organ.

**Supplementary Information:**

The online version contains supplementary material available at 10.1007/s10265-024-01543-2.

## Introduction

The stem is the organ that defines erected vascular plants, and hence their vegetative and reproductive functions. Inflorescence stems in most flowering plants are cylindrical in shape, grow upward, and are self-supported vertically while being flexible enough to manage internal and external fluctuations (Moulia et al. 2021). Classically, the stem has been investigated for its physiological and mechanical properties as well as its cell wall components (Kutschera and Niklas 2007; Trinh et al. 2021). However, determining the average cell wall stiffness alone is not sufficient to understand the mechanical properties of the stem at the organ level; rather, a multiscale view of the different contributions of cells and tissues to stem mechanics is critical.

The inflorescence stem of annuals has a radial symmetry, from a transverse section standpoint. The stem of *Arabidopsis thaliana* (L.) Heynh. (hereafter referred to as Arabidopsis) plants is composed of parenchyma-xylem-parenchyma tissues from the outside to the inside. Outer parenchyma contains epidermal, cortical, and endodermal cells, whereas inner parenchyma is composed mainly of pith cells, and occupies a larger volume than the outer parenchyma. Xylem is composed of fiber cells and vascular bundles. While the primary cell wall is present in all plant cell types, the secondary cell wall (SCW) is formed on the inner side of the primary cell wall only in fiber cells and vessel cells. The SCW contains lignin, which physically reinforces the wall, thereby contributing to increased global strength at the organ level (Ragni and Greb 2018). Although SCW performs essential mechanical and hydraulic functions in trees to support their large biomass (Badel et al. 2015), it also plays a substantial role in annuals. For example, Arabidopsis mutants lacking SCW synthesis capacity in vessel cells exhibit lethal growth defects, while those exhibiting reduced SCW synthesis in fiber cells display typical pendant stem phenotypes (Meester et al. 2018; Mitsuda et al. 2007; Sakamoto et al. 2018).

Recent progress in stem mechanics, and more specifically in the relative contributions of tissue layers, have emerged at the crossroads with molecular genetics. In Arabidopsis lines, imbalanced growth between the outer and inner tissues of the inflorescence stem triggers cracks in the epidermis and sometimes leads to organ breakage (Asaoka et al. 2021, 2023; Hentrich et al. 2013; Maeda et al. 2014). The stem cracking phenotype is caused by the presence of substantial differences in elastic strain between the epidermis and inner tissues (Baskin and Jensen 2013). This is especially notable in the *clavata3-8 de-etiolated3-1* (*clv3-8 det3-1*): the *clv3-8* mutation enhances cell proliferation (Gruel et al. 2016; Laufs et al. 1998; Reddy and Meyerowitz 2005), while the *det3-1* mutation decreases levels of cellulose synthesis by impairing the intracellular trafficking of cellulose synthase complexes (Canõ-Delgado et al. 2003; Luo et al. 2015; Padmanaban et al. 2004; Schumacher et al. 1999). The mechanical properties of epidermal cells in *det3-1* and *clv3-8 det3-1* stems were confirmed to be altered by the indentation method using atomic force microscopy (Asaoka et al. 2021). This cracking phenotype is consistent with the epidermal growth theory, where the epidermis is the load-bearing tissue (under tension) and thus limits growth (Asaoka et al. 2021; Galletti et al. 2016; Kutschera and Niklas 2007).

A recent study also highlighted more complex contributions of inner tissues; genetically increased expansion of inner non-lignified interfascicular cells through genetic means was sufficient to stretch the epidermis and induce cracks (Asaoka et al. 2023). Simultaneously, this result questions the role of inner tissues in the cracking phenotype of the *clv3-8 det3-1* double mutant. In particular, because stems of the *clv3-8 det3-1* double mutant exhibit altered inner morphology, we hypothesize that the unorganized distribution of structural components in the *clv3-8 det3-1* stem leads to a mechanically weak structure that fails to maintain structural integrity and finally breaks.

## Materials and methods

### Plant materials

Arabidopsis, Columbia (Col-0) accession was used as the wild-type (WT). Arabidopsis seeds were gas-sterilized with chlorine (4 mL of 37% HCl in 100 mL of bleach) for 3 h. The sterilized seeds were sown in pots filled with soil, and placed in a long-day growth chamber (16 h light, 21 °C, 150 μmol m^−2^ s^−1^). The following Arabidopsis lines were used in this study: *det3-1, clv3-8*, and *clv3-8 det3-1* (Maeda et al. 2014); *pPDF1:DET3 clv3-8 det3-1* (Asaoka et al. 2021); *nst1-1 nst3-1* (Mitsuda et al. 2007); *clv3-8 det3-1 nst1-1 nst3-1* (Asaoka et al. 2023). The *clv3-8 det3-1 nst1-1 nst3-1* mutant was generated in this study by crossing *nst1-1 nst3-1* with *clv3-8 det3-1*. Mutants were selected visually based on their phenotypes, i.e., increased leaflet number (*clv3-8*) and dwarf stature (*det3-1*). The primers used to genotype *nst1-1 nst3-1* have been reporter previously (Mitsuda et al. 2007).

### Physical measurements

Stem segments (ca. 100 mm in length), including the base were cut from the plants at ca. 40 days after sowing, and stored at 4 °C in aqueous solutions containing differing percentages of ethanol (EtOH). To perform the bending test, stem segments (ca. 35 mm long) were prepared and immediately placed on two jaws with a spacing of 30 mm in a micro-testing machine (Deben, MT2000).

A central pushing jaw was used in the compression mode to apply a constant speed displacement (11.5–12.0 µm s^−1^). The resulting loading force and the displacement were recorded until the central part of the sample had moved by 2 mm. A force–elongation curve was plotted using a home-made Python3 pipeline. The first part of the curve, which was linear, was used to compute the flexural rigidity of the stem. Flexural rigidity (*EI,* N m^2^) was computed according to the following equation:

$$EI=\left(dF/dY\right){L}^{3}/48$$

where F is the applied force, Y is the displacement of central jaw; *L* is the distance between the external jaws; and *I* is the second moment of the stem cross-section.

Young’s modulus (*E*, Pa) was calculated by dividing the flexural rigidity (*EI*) with the second moment of the stem cross-section (*I*). Because the stem material is not homogeneous, we refer to the calculated Young’s modulus as “apparent elastic modulus”. Average stem diameter was obtained by measuring the diameter at the middle of the segment (at least three point measurements per stem) using a caliper. To determine the shape of non-cylindrical stems, cross-sections were prepared from the middle of the stem by hand sectioning. *I* was calculated depending on the shape of the stem cross-sections: as π*D*^4^/64 for circular cross-sections, *bh*^3^/12 for rectangular cross-sections, π*a*^3^*b*/64 for oval cross-sections, and *bh*^3^/36 for triangular cross-sections; where *D* is the stem diameter; *a* is the thickness of the sample parallel to the bending direction; *h* is the thickness of sample parallel to the bending direction (e.g., rectangular or triangular sections); and *b* is the width of the sample perpendicular to the bending direction.

In a few instances, to examine the effect of cracking on the strength of the whole stem, an artificial longitudinal crack was manually generated in the middle part of stem using a scalpel just before taking measurements. The crack was 20 mm long and was deep enough to reach approximately the center of the stem.

### Microscopy observations

Stem cross-sections prepared by hand were observed under a stereomicroscope (Zeiss Imager.M2 microscope equipped with a Axiocam 503 camera). The shape of stem cross-sections was determined using ImageJ. Since the images of stem cross-sections included trichomes, the outer perimeter of the stem devoid of trichomes was manually traced. Subsequently, the extracted images of the stem periphery were subjected to analysis with the following shape descriptors: area, perimeter, solidity, and circularity.

## Results

### Reduced stem flexural rigidity in *det3-1*

**Fig. 1:**
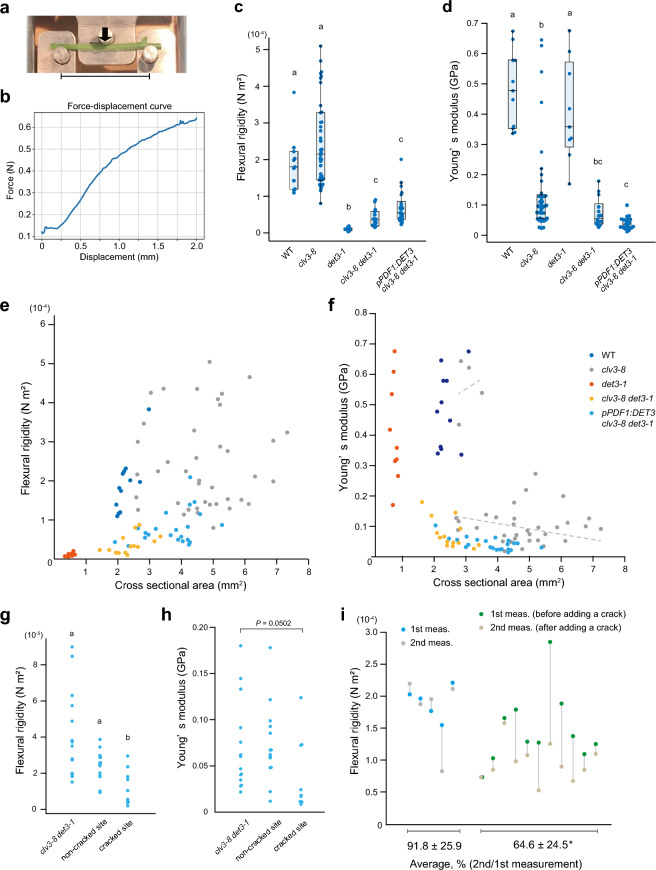
Three-point bending test on Arabidopsis stems*.* (**a**) Photograph showing the two outside jaws, separated by 30 mm, on which the sample is placed. Arrow indicates the direction of movement of the central jaw. (**b**) Typical force–displacement curve obtained by three-point bending test. (**c**, **d**) Results of the three-point bending test performed on Arabidopsis mutants. (**c**) Flexural rigidity. (**d**) Young’s modulus. Boxplots show quartiles (box limits) and medians (horizontal lines). Whiskers indicate the total range (minimum and maximum values). Different letters indicate significant differences among genotypes (Tukey’s test, *P* < 0.05). (**e**, **f**) Flexural rigidity (**e**) and Young’s modulus (**f**) plotted against the cross-sectional area of the stem segment. Plots show the results of the three-point bending test, based on stem size and shape. Correlation lines of the minor peak (upper line) or major peak (lower line) of Young’s modulus in *clv3-8* are indicated. (**g**, **h**) Flexural rigidity (**g**) and Young’s modulus (**h**) of the *clv3-8 det3-1* double mutant measured by the three-point bending test. The *clv3-8 det3-1* stem sample used in this experiment (left) had no cracked part. Different letters indicate significant differences (Tukey's test, *P* < 0.05). (**i**) Comparison between the first and second measurements taken during the three-point bending test performed on WT stems. The two values within a column represent the results obtained from the same stem. An asterisk indicates significant difference (Welch’s test, *P* < 0.05)

To go beyond the local measurement of outer epidermal wall stiffness with atomic force microscopy (AFM) and measure more global mechanical features of WT and *clv3-8 det3-1* stems, we used a three-points bending test in which the force versus bending was continuously recorded during the test. Flexural rigidity was deduced from the slope of the linear part of the force–displacement curve (Fig. 1a, b).

We carried out the three-point bending test on the stems of wild-type (WT), *clv3-8*, *det3-1* and the double mutant *clv3-8 det3-1* (exhibiting stem cracking). We also examined the *pPDF1:DET3 clv3-8 det3-1* line, in which the cracking frequency was significantly reduced (Asaoka et al. 2021). The *pPDF1:DET3* gene genotype is exclusively expressed in the epidermal tissue in-specific complementation line of *clv3-8 det3-1* background, and its reduced crack frequency has been reported previously (Asaoka et al. 2021). The tests were conducted using stem portions prepared from the first internode of the main inflorescence stem of WT and *clv3-8* plants, from most of the stems of *det3-1* plants (since they were severely dwarf), and from the stem portions (avoiding the cracked parts) of *clv3-8 det3-1* and *pPDF1:DET3 clv3-8 det3-1* plants*.*

The flexural rigidity of WT stems was 1.89 ± 0.74 × 10^–4^ N m^2^, which was comparable with that of *clv3-8* (2.46 ± 1.17 × 10^–4^ N m^2^), suggesting that reduced cell differentiation was compensated by thicker stem in the *clv3-8* background (Fig. 1c). However, the *det3-1*, *clv3-8 det3-1*, and *pPDF1:DET3 clv3-8 det3-1* stems exhibited one order of magnitude lower flexural rigidity than that of the WT (0.100 ± 0.05 × 10^–4^ N m^2^; 0.415 ± 0.24 × 10^–4^ N m^2^; 0.683 ± 0.43 × 10^–4^ N m^2^, respectively; Fig. 1c). Because the forces detected in *det3-1* were close to the lowest limit of detection by the force cell, most probably because the stems were very thin, we cannot exclude the possibility that the resolution of the technique did not allow us to obtain valid values for *det3-1*. On the other hand, the *pPDF1:DET3 clv3-8 det3-1* line displayed higher flexural rigidity than *det3-1*, consistent with partial complementation in the epidermis. This also revealed that the impact of the *det3-1* mutation on flexural rigidity does not depend on the presence of the *clv3-8* mutation. Yet flexural rigidity is in essence very integrative, and may not reflect the root cause of stiffness defects. This is what we analyzed next.

### Reduced stem stiffness in *clv3-8*

Flexural rigidity provides a global mechanical response, but it is not normalized by stem geometry. Theoretically, when the stem material properties are conserved, the flexural rigidity of the stem increases with the increase in its thickness. The three-point bending test provides the Young’s modulus (apparent elastic modulus), which measures the stiffness of a material and is calculated by dividing the stem flexural rigidity with the second moment of inertia of an area, namely, the size and/or shape of the cross-section of the bending material (Fig. 1b).

The stiffness of WT stem was 0.482 ± 0.125 GPa, which is within the same range as the previously reported values for Arabidopsis stems (ex. MacMillan et al. 2010; Timpano et al. 2015). The Young’s modulus of *det3-1* stems (0.407 ± 0.167 GPa) was comparable to that of the WT. This suggests that, despite the presence of weaker cell walls in the epidermis (as previously measured by AFM), the bulk of the *det3-1* stem is as stiff as the WT, and the reduced flexural rigidity of the *det3-1* stem is rather caused by a reduction in its cross-sectional size (Fig. 1d). A significant reduction in Young’s modulus was observed in *clv3-8* (0.146 ± 0.156 GPa), *clv3-8 det3-1* (0.074 ± 0.047 GPa) and *pPDF1:DET3 clv3-8 det3-1* (0.040 ± 0.020 GPa), pointing toward a dominant contribution of *clv3-8* in this response (Fig. 1d). This could be explained by the reduced level of differentiation in *clv3-8* (where cell proliferation is favored instead).

To further analyze these quantifications, the measurements of flexural rigidity and Young’s modulus were plotted against the cross-sectional area of the stem in order to further analyze these quantifications (Fig. 1e, f). The results revealed a high degree of heterogeneity in the Young’s modulus of *det3-1* stems, which may be attributed to the resolution of the technique and to the difficulty in characterizing the real shape of the cross-section. Yet, the distribution confirmed the remarkable impact of *pPDF1:DET3 det3-1* on Young’s modulus and flexural rigidity (when compared with *det3-1*). Despite the increased cross-sectional area of the *clv3-8* stem, its flexural rigidity was comparable with that of the WT. The Young’s modulus of *clv3-8* stem seemed to show a bimodal response, with a minor peak comparable with the WT (approximately 0.5 GPa; Fig. 1f, upper line) and a major peak lower than the WT (approximately 0.1 GPa; Fig. 1f, lower line). This confirms that the presence of a fasciated and wider stem, along with reduced cell differentiation, in *clv3-8* does not majorly impact flexural rigidity but affects the Young’s modulus. The Young’s modulus of *clv3-8 det3-1* was approximately 0.1 GPa, with no outliers in the 0.5 GPa, suggesting that both mutations have a synergistic impact on Young’s modulus.

### Longitudinal cracking in *clv3-8 det3-1* reduces flexural rigidity

Next, we focused on the effect of cracking on the strength of the whole stem. We used the *clv3-8 det3-1* stems with cortical cracks (but no complete breaks) and applied force to the cracked part or non-cracked parts. During force loading on a non-cracked part of the stem, a cracked part of the stem appeared closed, and behaved like a cylinder. The results indicated that flexural rigidity decreased when bending the cracked part (*clv3-8 det3-1*: 2.40 ± 0.83 × 10^–5^ N m^2^ at the non-cracked site vs. 1.17 ± 0.96 × 10^–5^ N m^2^ at the cracked site; Fig. 1g). The non-cracked part of the *clv3-8 det3-1* stem showed no change in Young’s modulus (0.074 ± 0.047 GPa at the non-cracked site vs. 0.076 ± 0.040 GPa at the non-cracked part of the cracked stem) (Fig. 1h). This is consistent with a scenario in which local cracking does not affect the material properties beyond the cracked site.

To further confirm the global impact of cracks on the flexural rigidity of the stem, we artificially induced a single longitudinal crack in WT stems and tested the hysteresis of the stem by repeating the load application. The first and second measurements recorded on the same stem provided similar results (Fig. 1i). Then, we carried out the bending test twice on the same sample, once before and once after we created the artificial crack. These results indicated that the longitudinal crack in the stem led to the loss of whole-stem rigidity, confirming that structural continuity provides strength to the stem (Fig. 1i).

### *clv3-8-*dependent reduction in stem stiffness does not depend on stem cross-section shape

**Fig. 2:**
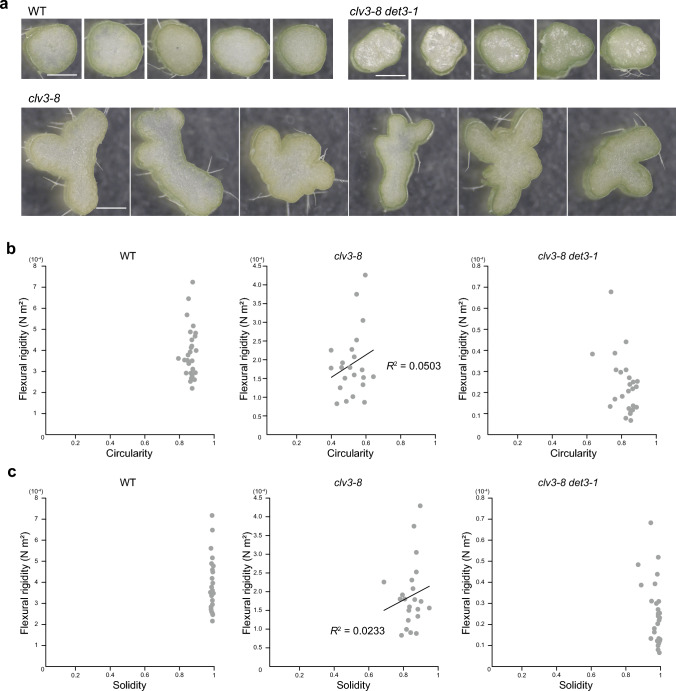
Quantification of the stem shape of *clv3-8.* (**a**) Representative images of stem cross-sections. Scale bar = 100 µm. Samples were prepared by manual cutting after the bending test. (**b**, **c**) Circularity (**b**) and solidity (**c**) of stem cross-sections plotted against stem cross-sectional area

Next, we quantified the shape of the WT, *clv3-8*, and *clv3-8 det3-1* stems and determined the correlation between stem shape and mechanical properties. The cross-sections of *clv3-8* stems showed variable shapes (Fig. 2a). The circularity and solidity of stem cross-sections were almost constant in the WT but varied considerably in *clv3-8*. Yet, stem circularity and solidity showed no simple correlation with flexural rigidity in *clv3-8* (Fig. 2b), suggesting that other factors, such as reduced cell differentiation, may be responsible for the reduction in material rigidity. Although circularity and solidity are classic parameters to define object shape contours, they might not be the most relevant to express real shape of stem cross-sections of *clv3-8*. In particular, local shape deviations in stem sections may have a strong global effect on stem mechanics. For instance, the *clv3-8 det3-1* stem cross-sections were almost circular in shape, but some stem cross-sections were more rectangular than circular (Fig. 2b).

Altogether, we found that the fasciation observed in the *clv3-8* stem is markedly abolished by the *det3-1* mutation, and that changes in the shape of *clv3-8* stem cross-sections are not sufficient to explain the reduction in Young’s modulus.

### Lignified tissue and its organization are required for robust stem structure

To analyze the extent of cell differentiation in *clv3-8*, *det3-1* and *clv3-8 det3-1*, we analyzed the identity of stem layers, using astra blue/safranin double staining. We quantified the area occupied by parenchyma tissue (outer or inner) or lignified tissue within the stem cross-section. Microscopy observations revealed that the inner parenchyma tissue occupied a greater area in the *clv3-8* stem than in the WT stem (Fig. S1a, b). Note that this difference in parenchyma tissue area between *clv3-8* and WT occurred, despite the increased number of vascular bundles in *clv3-8*. Additionally, the percentage of lignified area varied slightly among genotypes (approximately 25% in WT, 20% in *clv3-8*, and 23% in *clv3-8 det3-1*). In *clv3-8 det3-1*, the medullar parenchyma exhibited large cells.

**Fig. 3:**
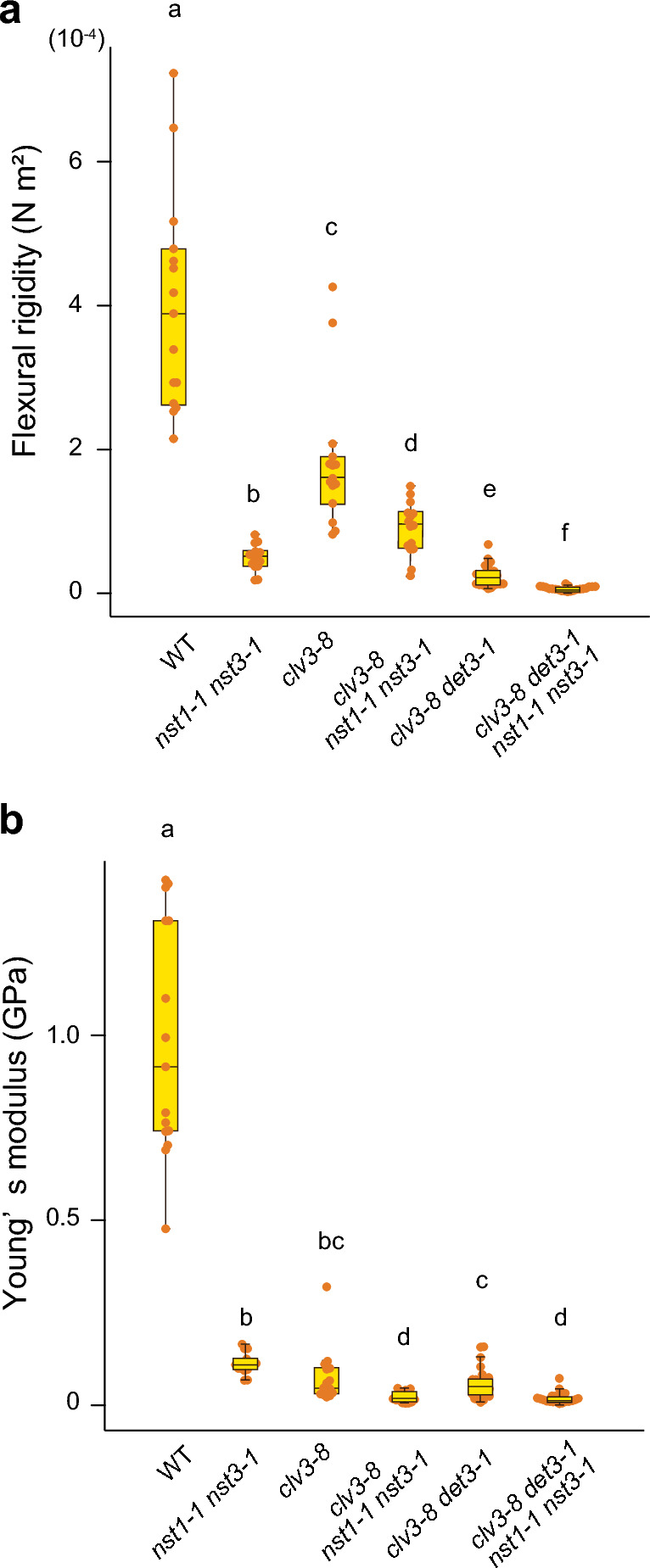
Results of three-point bending test performed on mutant stems without lignified fiber tissue. (**a**, **b**) Flexural rigidity (**a**) and Young’s modulus (**b**) calculated from the three-point bending test. Boxplots show quartiles (box limits) and medians (horizontal lines). Whiskers indicate the total range (minimum and maximum values). Different letters indicate significant differences among genotypes (Tukey's test, *P* < 0.05)

To test the contribution of lignification to the reduction in Young’s modulus in *clv3-8 det3-1*, we analyzed mutants with lignification defects. The *nst1-1 nst3-1* double mutant lacked lignified fiber cells and showed a pendant stem phenotype (Mitsuda et al. 2007). Stem posture and whole-stem stiffness were related. However, considering that the *nst1-1 nst3-1* stem could stand upright at the early stages of growth, the contribution of *NST1* and *NST3* gene genotypes to stem stiffness remains unclear. The stem cross-sections of both *nst1-1 nst3-1* and the WT were almost circular in shape (mean value of circularity in *nst1-1 nst3-1* = 0.847 ± 0.068), and their area was similar (Fig. S1a). The three-point bending test described above revealed that flexural rigidity was one order of magnitude lower (-87%) in *nst1-1 nst3-1* (4.95 ± 1.77 × 10^–5^ N m^2^) than in the WT (4.00 ± 1.45 × 10^–4^ N m^2^) (Fig. 3a).

Next, we generated higher-order mutants by crossing *nst1-1 nst3-1*, c*lv3-8*, and *clv3-8 det3-1* in various combinations, and performed the three-point bending test. The results showed that the flexural rigidity of *clv3-8 nst1-1 nst3-1* (8.99 ± 3.65 × 10^–5^ N m^2^) and *clv3-8 det3-1 nst1-1 nst3-1* (6.88 ± 2.94 × 10^–6^ N m^2^) stems was lower than that of *nst1-1 nst3-1* stems (4.95 ± 1.77 × 10^–5^ N m^2^) (Fig. 3a).

**Fig. 4:**
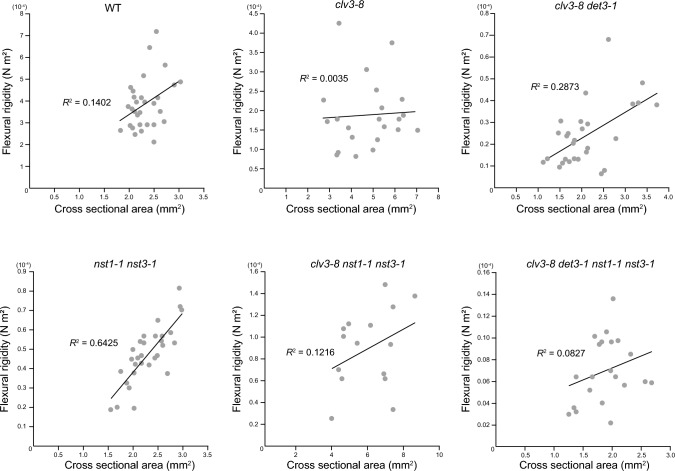
Result of the three-point bending test based on the size of stem cross-sections. Correlations between stem cross-sectional area and flexural rigidity were calculated

**Fig. 5:**
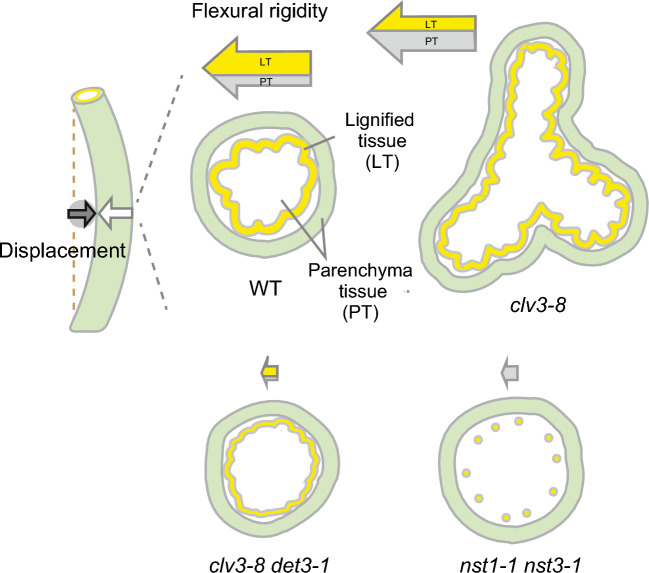
Schematic model showing the structural contribution of stem tissues to organ rigidity. In the WT, lignified tissue is a wavy and hollow cylinder that contains fiber cells and vessel cells. Lignified tissue occupies 25% of stem organ and contributes to approximately 80% of stem rigidity against bending. In the *nst1-1 nst3-1* mutant, only the vessel cells are lignified, and they together act as a pillar. Increase in parenchyma tissue contributes linearly to flexural rigidity in *nst1-1 nst3-1*, unlike that in the WT. In *clv3-8*, the stem is a star-shaped polygon and has 2–threefold greater volume than the WT stem. Despite the greater structural volume, the flexural rigidity of the *clv3-8* stem is not as high as that of the WT stem. In *clv3-8 det3-1,* the stem is a cylindrical structure containing lignified tissue, in which vascular bundles appear as narrow waves and are higher in number than those in the WT stem. Despite the presence of lignified tissue in the *clv3-8 det3-1* stem, its contribution to flexural rigidity is not significant. The size of the arrows corresponds to flexural rigidity, with the estimated contributions of each parenchyma tissue (PT) and lignified tissue (LT) indicated

Note that the positive correlation between cross-sectional area and flexural rigidity could also be confirmed in *nst1-1 nst3-1* by analyzing the distribution of these values (Fig. 4). The increase in cross-sectional area was linearly associated with higher flexural rigidity in *nst1-1 nst3-1*, but this correlation was not observed in the other lines tested (Fig. 4). These results suggest that an increase in stem parenchyma tissue leads to a minimal increase in flexural rigidity, while the lignification of inner tissues leads to a substantial increase in flexural rigidity. Considering that the difference in flexural rigidity between *clv3-8* and *clv3-8 nst1-1 nst3-1* (Fig. 3a), although statistically significant, was not drastic, the rigidity conferred by lignified tissue to a polygonal structure was lower than that conferred to a cylindrical structure. This difference was also observed between *clv3-8 det3-1* and *clv3-8 det3-1 nst1-1 nst3-1*. Taken together, this suggests that proper distribution of lignified tissue is required to provide structural support to the inflorescence stem (Fig. 5).

The values of Young’s modulus in *clv3-8 nst1-1 nst3-1* (0.020 ± 0.014 GPa) and *clv3-8 det3-1 nst1-1 nst3-1* (0.020 ± 0.015 GPa) were lower than that in *nst1-1 nst3-1* (0.111 ± 0.028 GPa) (Fig. 3b). This further confirms the dominant role of lignification in the material properties of the stem. Although a clear correlation between cross-sectional area and Young’s modulus was not observed in all lines, a negative correlation between these two variables was detected in WT, *clv3-8 nst1-1 nst3-1*, and *clv3-8 det3-1* (Fig. S2). This result indicates that these stem structures are less mechanically effective for their volume.

## Discussion

The bending test is widely used to study the mechanical properties of wood, which has greater rigidity than most soft tissues of plants (Shah et al. 2017). Although the bending test has not been applied extensively to the Arabidopsis stem, cellulose-deficient mutants or irregular xylem mutants display reduced bending stiffness (Amanda et al. 2017; Bichet et al. 2001; Jones et al. 2001; Meester et al. 2018; Turner and Somerville 1997; Zhong et al. 1997). Our study demonstrates that cylindrical structure, structural continuity, and lignified fiber tissue are essential for the stiffness and flexural rigidity of the Arabidopsis stem. Stem rigidity in the *clv3-8 det3-1* stem cracking mutant decreased (Fig. 1c). In a previous study, the mechanical properties of the cell wall were reduced in both *det3-1* and *clv3-8 det3-1* (Asaoka et al. 2021). Therefore, the factor that affects whole-stem mechanical properties was expected to be the mutational effect of *det3-1*. However, our results indicate that the whole-stem mechanical properties are affected by the *clv3-8* mutation (Fig. 1c, d). Although the *det3-1* stem was thin, and its measurements approached the detection limit of the force cell, the results obtained from some of the *det3-1* stems indicated that material properties, as represented by Young’s modulus, are not compromised in *det3-1,* unlike in *clv3-8* (Fig. 1c). *CLV3* is preferentially expressed in the shoot apical meristem and not in mature tissues, such as the basal part of the stem, which was used in this study (ex. Fuchs and Lohmann 2020). Therefore, long-distance impact of enhanced cell proliferation, i.e. with a relative reduction in cell differentiation/lignification might result in a stem with low structural rigidity.

Although lignified tissue occupies only ~ 25% of the stem cross-sectional area, our study revealed its significant contribution to stem stiffness. The level of lignification can be altered either by tapping into the lignification process (*NST*) or by altering the ratio of cell proliferation to cell differentiation (*clv3*). The decrease of stiffness, force/extension along to the longitudinal direction in this case, was also confirmed in the *nst1-1 nst3-1* stem by tensile tests as well (Sakamoto et al. 2018). At the nano-scale, lignified cell walls were indeed found to exhibit higher stiffness thorough the nano-indentation method by AFM (Asgari et al. 2020; Brulé et al. 2019; Ménard et al. 2022). Nevertheless, at the organ scale, a correlation between cross-sectional area and flexural rigidity was observed in the *nst1-1 nst3-1* stem, but not in the WT, indicating that stem flexural rigidity becomes more complex when tissue layers exhibit lignification (Fig. 4). This is probably due to the complex spatial distribution of lignified inner tissue in stem. No correlation between cross-sectional area and flexural rigidity was observed in non-circular *clv3-8 nst1-1 nst3-1* stem (Fig. 4). Note that even though *nst1-1 nst3-1* lacks SCW in fiber cells (Mitsuda et al. 2007; Fig. S1), and exhibits similar cross-sectional area, the flexural rigidity and Young’s modulus were higher in *nst1-1 nst3-1* than in *clv3-8 det3-1*. This again highlights the reduction of stem rigidity in the *clv3-8* background. These results suggest that reinforcement by lignified tissue in *nst1-1 nst3-1* is not of the same magnitude as that in the WT.

Stem cross-sectional shape was not constant and showed no correlation with flexural rigidity in the *clv3-8* mutant, indicating that there may be other factors that affect the stem stiffness. The thicker stem of *clv3-8* is associated with an increase in vascular bundle number. This increase in vascular bundle number alters the pattern of stem cracking observed in other stem cracking mutants (Asaoka et al. 2023). The results of the current study imply that the increase in vascular bundle number reduces stem rigidity. However, whether the total number of vascular bundles or the associated changes in the distribution pattern of fiber tissue alter the mechanical properties of the organ remains to be investigated. In summary, *clv3-8 det3-1* has a weaker stem than the WT, which is most likely caused by the altered organization of lignified tissue. Both the presence of lignified tissue and its proper alignment within the stem are essential for conferring higher rigidity to the stem.

## Conclusions and future prospects

In conclusion, our results demonstrated that bending tests are a good approach for investigating the mechanical properties of stems. In the future, a highly sensitive force cell should be used to study the Arabidopsis stem. Determining the real shape of stem cross-sections would be important for obtaining a more precise measurement of the second moment “*I*”. Our results showed that the localization of different tissues plays a key role in determining the mechanical behavior of the stem. Further studies, for example, using other mutants or plants grown under different environmental conditions, would be necessary to confirm these results.

## Supplementary Information

Below is the link to the electronic supplementary material.

Supplementary file1 (PDF 1407 KB)

## Data Availability

The data that support the findings of this study are available from the corresponding author upon reasonable request.

## References

[CR1] Amanda D, Doblin MS, MacMillan CP et al (2017) Arabidopsis DEFECTIVE KERNEL1 regulates cell wall composition and axial growth in the inflorescence stem. Plant Direct 1:e00027. 10.1002/pld3.27 31245676 10.1002/pld3.27 PMC6508578

[CR2] Asaoka M, Ooe M, Gunji S et al (2021) Stem integrity in *Arabidopsis thaliana* requires a load-bearing epidermis. Development 148:dev198028. 10.1242/dev.198028 10.1242/dev.198028 33637612

[CR3] Asaoka M, Sakamoto S, Gunji S, Mitsuda N, Tsukaya H, Sawa S, Hamant O, Ferjani A (2023) Contribution of vasculature to stem integrity in *Arabidopsis thaliana.* Development 150:dev201156. 10.1242/dev.201156 10.1242/dev.201156 36746191

[CR4] Asgari M, Brulé V, Western TL et al (2020) Nano-indentation reveals a potential role for gradients of cell wall stiffness in directional movement of the resurrection plant *Selaginella lepidophylla*. Sci Rep 10:506. 10.1038/s41598-019-57365-z 31949232 10.1038/s41598-019-57365-z PMC6965169

[CR5] Badel E, Ewers FW, Cochard H, Telewski FW (2015) Acclimation of mechanical and hydraulic functions in trees: impact of the thigmomorphogenetic process. Front Plant Sci 6:266. 10.3389/fpls.2015.00266 25954292 10.3389/fpls.2015.00266 PMC4406077

[CR6] Baskin TI, Jensen OE (2013) On the role of stress anisotropy in the growth of stems. J Exp Bot 64:4697–4707. 10.1093/jxb/ert176 10.1093/jxb/ert176 23913952

[CR7] Bichet A, Desnos T, Turner S et al (2001) *BOTERO1* is required for normal orientation of cortical microtubules and anisotropic cell expansion in Arabidopsis. Plant J 25:137–148. 10.1111/j.1365-313x.2001.00946.x 11169190 10.1046/j.1365-313x.2001.00946.x

[CR8] Brulé V, Rafsanjani A, Asgari M, Western TL, Pasini D (2019) Three-dimensional functional gradients direct stem curling in the resurrection plant *Selaginella lepidophylla*. J R Soc Interface 16:20190454. 10.1098/rsif.2019.0454 31662070 10.1098/rsif.2019.0454 PMC6833318

[CR9] Canõ-Delgado A, Penfield S, Smith C, Catley M, Bevan M (2003) Reduced cellulose synthesis invokes lignification and defense responses in *Arabidopsis thaliana*. Plant J 34:351–362. 10.1046/j.1365-313X.2003.01729.x 12713541 10.1046/j.1365-313x.2003.01729.x

[CR10] Fuchs M, Lohmann JU (2020) Aiming for the top: non-cell autonomous control of shoot stem cells in Arabidopsis. J Plant Res 133:297–309. 10.1007/s10265-020-01174-3 32146616 10.1007/s10265-020-01174-3 PMC7214502

[CR11] Galletti R, Verger S, Hamant O, Ingram GC (2016) Developing a ‘thick skin’: a paradoxical role for mechanical tension in maintaining epidermal integrity? Development 143:3249–3258. 10.1242/dev.132837 27624830 10.1242/dev.132837

[CR12] Gruel J, Landrein B, Tarr P, Schuster C, Refahi Y, Sampathkumar A, Hamant O, Meyerowitz EM, Jönsson H (2016) An epidermis-driven mechanism positions and scales stem cell niches in plants. Sci Adv 2:e1500989. 10.1126/sciadv.1500989 10.1126/sciadv.1500989 PMC4846443 27152324

[CR13] Hentrich M, Sánchez-Parra B, Alonso M-MP et al (2013) *YUCCA8* and *YUCCA9* overexpression reveals a link between auxin signaling and lignification through the induction of ethylene biosynthesis. Plant Signal Behav 8:e26363. 10.4161/psb.26363 24022251 10.4161/psb.26363 PMC4106514

[CR14] Jones L, Ennos AR, Turner SR (2001) Cloning and characterization of irregular *xylem4* (*irx4*): a severely lignin-deficient mutant of Arabidopsis. Plant J 26:205–216. 10.1046/j.1365-313x.2001.01021.x 11389761 10.1046/j.1365-313x.2001.01021.x

[CR15] Kutschera U, Niklas KJ (2007) The epidermal-growth-control theory of stem elongation: an old and a new perspective. J Plant Physiol 164:1395–1409. 10.1016/j.jplph.2007.08.002 17905474 10.1016/j.jplph.2007.08.002

[CR16] Laufs P, Grandjean O, Jonak C, Kiêu K, Traas J (1998) Cellular parameters of the shoot apical meristem in Arabidopsis. Plant Cell 10:1375–1390. 10.2307/3870647 9707536 10.1105/tpc.10.8.1375 PMC144064

[CR17] Luo Y, Scholl S, Doering A et al (2015) V-ATPase activity in the TGN/EE is required for exocytosis and recycling in Arabidopsis. Nat Plants 1:15094. 10.1038/nplants.2015.94 27250258 10.1038/nplants.2015.94 PMC4905525

[CR18] MacMillan CP, Mansfield SD, Stachurski ZH, Evans R, Southerton SG (2010) Fasciclin-like arabinogalactan proteins: Specialization for stem biomechanics and cell wall architecture in Ara- bidopsis and Eucalyptus. Plant J 62:689–703. 10.1111/tpj.2010.62.issue-4 20202165 10.1111/j.1365-313X.2010.04181.x

[CR19] Maeda S, Gunji S, Hanai K, Hirano T, Kazama Y, Ohbayashi I, Abe T, Sawa S, Tsukaya H, Ferjani A (2014) The conflict between cell proliferation and expansion primarily affects stem organogenesis in Arabidopsis. Plant Cell Physiol 55:1994–2007. 10.1093/pcp/pcu131 25246492 10.1093/pcp/pcu131

[CR20] Meester BD, de Vries L, Özparpucu M et al (2018) Vessel-specific reintroduction of CINNAMOYL-COA REDUCTASE1 (CCR1) in dwarfed *ccr1* mutants restores vessel and xylary fiber integrity and increases biomass. Plant Physiol 176:611–633. 10.1104/pp.17.01462 29158331 10.1104/pp.17.01462 PMC5761799

[CR21] Ménard D, Blaschek L, Kriechbaum K, Lee CC, Serk H, Zhu C, Lyubartsev A, Nuoendagula N, Bacsik Z, Bergström L et al (2022) Plant biomechanics and resilience to environmental changes are controlled by specific lignin chemistries in each vascular cell type and morphotype. Plant Cell 34:4877–4896. 10.1093/plcell/koac284 36215679 10.1093/plcell/koac284 PMC9709985

[CR22] Mitsuda N, Iwase A, Yamamoto H et al (2007) NAC transcription factors, NST1 and NST3, are key regulators of the formation of secondary walls in woody tissues of Arabidopsis. Plant Cell 19:270–280. 10.1105/tpc.106.047043 17237351 10.1105/tpc.106.047043 PMC1820955

[CR23] Moulia B, Douady S, Hamant O (2021) Fluctuations shape plants through proprioception. Science 372:. 10.1126/science.abc6868 10.1126/science.abc6868 33888615

[CR24] Padmanaban S, Lin X, Perera I, Kawamura Y, Sze H (2004) Differential expression of vacuolar H+-ATPase subunit c genes in tissues active in membrane trafficking and their roles in plant growth as revealed by RNAi. Plant Physiol 134:1514–1526. 10.1104/pp.103.034025 15051861 10.1104/pp.103.034025 PMC419827

[CR25] Ragni L, Greb T (2018) Secondary growth as a determinant of plant shape and form. Semin Cell Dev Biol 79:58–67. 10.1016/j.semcdb.2017.08.050 28864343 10.1016/j.semcdb.2017.08.050

[CR26] Reddy GV, Meyerowitz EM (2005) Stem-cell homeostasis and growth dynamics can be uncoupled in the Arabidopsis shoot apex. Science 310:663–667. 10.1126/science.1116261 16210497 10.1126/science.1116261

[CR27] Sakamoto S, Somssich M, Nakata MT et al (2018) Complete substitution of a secondary cell wall with a primary cell wall in Arabidopsis. Nat Plants 4:777–783. 10.1038/s41477-018-0260-4 30287954 10.1038/s41477-018-0260-4

[CR28] Schumacher K, Vafeados D, McCarthy M, Sze H, Wilkins T, Chory J (1999) The Arabidopsis *det3* mutant reveals a central role for the vacuolar H^+^–ATPase in plant growth and development. Genes Dev 13:3259–3270. 10.1101/gad.13.24.3259 10617574 10.1101/gad.13.24.3259 PMC317205

[CR29] Shah DU, Reynolds TP, Ramage MH (2017) The strength of plants: theory and experimental methods to measure the mechanical properties of stems. J Exp Bot 68:4497–4516. 10.1093/jxb/erx245 28981787 10.1093/jxb/erx245

[CR30] Timpano H, Sibout R, Devaux MF et al (2015) *Brachypodium* cell wall mutant with enhanced saccharification potential despite increased lignin content. Bioenerg Res 8:53–67. 10.1007/s12155-014-9501-1

[CR31] Trinh D-C, Alonso-Serra J, Asaoka M et al (2021) How mechanical forces shape plant organs. Curr Biol 31:R143–R159. 10.1016/j.cub.2020.12.001 33561417 10.1016/j.cub.2020.12.001

[CR32] Turner SR, Somerville CR (1997) Collapsed xylem phenotype of Arabidopsis identifies mutants deficient in cellulose deposition in the secondary cell wall. Plant Cell 9:689–701. 10.1105/tpc.9.5.689 9165747 10.1105/tpc.9.5.689 PMC156949

[CR33] Zhong R, Taylor JJ, Ye Z-H (1997) Disruption of interfascicular fiber differentiation in an Arabidopsis mutant. Plant Cell 9:2159. 10.2307/3870576 9437861 10.1105/tpc.9.12.2159 PMC157065

