# Correction to: Contributions of lignification, tissue arrangement patterns, and cross-sectional area to whole-stem mechanical properties in *Arabidopsis thaliana*

**DOI:** 10.1007/s10265-025-01632-w

**Published:** 2025-04-07

**Authors:** Mariko Asaoka, Eric Badel, Ali Ferjani, Kazuhiko Nishitani, Olivier Hamant

**Affiliations:** 1 Laboratoire de Reproduction Et Développement Des Plantes, Université de Lyon, UCB Lyon 1, ENS de Lyon, INRAE, CNRS, 46 Allée d’Italie, 69364 Lyon Cedex 07, France; 2 Department of Biological Sciences, Faculty of Science, Kanagawa University, Yokohama-Shi, Kanagawa 221-8686 Japan; 3 Department of Biology, Tokyo Gakugei University, Koganei-Shi, Tokyo, 184-8501 Japan; 4 Université Clermont Auvergne, INRAE, PIAF, 63000 Clermont–Ferrand, France

**Correction to: Journal of Plant Research**

10.1007/s10265-024-01543-2

The article “Contributions of lignification, tissue arrangement patterns, and cross-sectional area to whole-stem mechanical properties in *Arabidopsis thaliana*”, written by Mariko Asaoka, Eric Badel, Ali Ferjani, Kazuhiko Nishitani and Olivier Hamant, was originally published under exclusive license to The Author(s) under exclusive licence to The Botanical Society of Japan. As a result of the subsequent decision to publish the article under the open access model, the article’s copyright notice was changed on 15 March 2025 to © The Author(s) 2025 and the article is now distributed under a Creative Commons Attribution 4.0 International License, which permits use, sharing, adaptation, distribution and reproduction in any medium or format, as long as you give appropriate credit to the original author(s) and the source, provide a link to the Creative Commons licence, and indicate if changes were made. The images or other third party material in this article are included in the article’s Creative Commons licence, unless indicated otherwise in a credit line to the material. If material is not included in the article’s Creative Commons licence and your intended use is not permitted by statutory regulation or exceeds the permitted use, you will need to obtain permission directly from the copyright holder. To view a copy of this licence, visit http://creativecommons.org/licenses/by/4.0.

The original article has been corrected.

