# Supplementary material for: Contributions of lignification, tissue arrangement patterns, and cross-sectional area to whole-stem mechanical properties in Arabidopsis thaliana
Source: J Plant Res. 2024 Apr 26;137(5):773–83. doi: 10.1007/s10265-024-01543-2 (PMC11976825; doi:10.1007/s10265-024-01543-2)
Supplement: Supplementary file 1 — Supplementary file1 (PDF 1407 KB) [file 10265_2024_1543_MOESM1_ESM.pdf]

**Article Title:**

# Contributions of lignification, tissue arrangement patterns, and cross-sectional area to whole-stem mechanical properties in *Arabidopsis thaliana*

**Journal name:**
Journal of Plant Research

**Author:**
Mariko Asaoka[1,2,3,*], Eric Badel[4], Ali Ferjani[3], Kazuhiko Nishitani[2], Olivier Hamant[1]

**Affiliation:**
[1] Laboratoire de Reproduction et Développement des Plantes, Université de Lyon, UCB Lyon 1, ENS de Lyon, INRA, CNRS, 46 Allée d'Italie, 69364 Lyon Cedex 07, France
[2] Department of Biological Sciences, Faculty of Science, Kanagawa University, Yokohama-shi, Kanagawa, 221-8686, Japan
[3] Department of Biology, Tokyo Gakugei University, Koganei-shi, Tokyo 184-8501, Japan
[4] Université Clermont Auvergne, INRAE, PIAF, 63000 Clermont–Ferrand, France

*Corresponding author for Contact:
Mariko Asaoka
asaoka@kanagawa-u.ac.jp

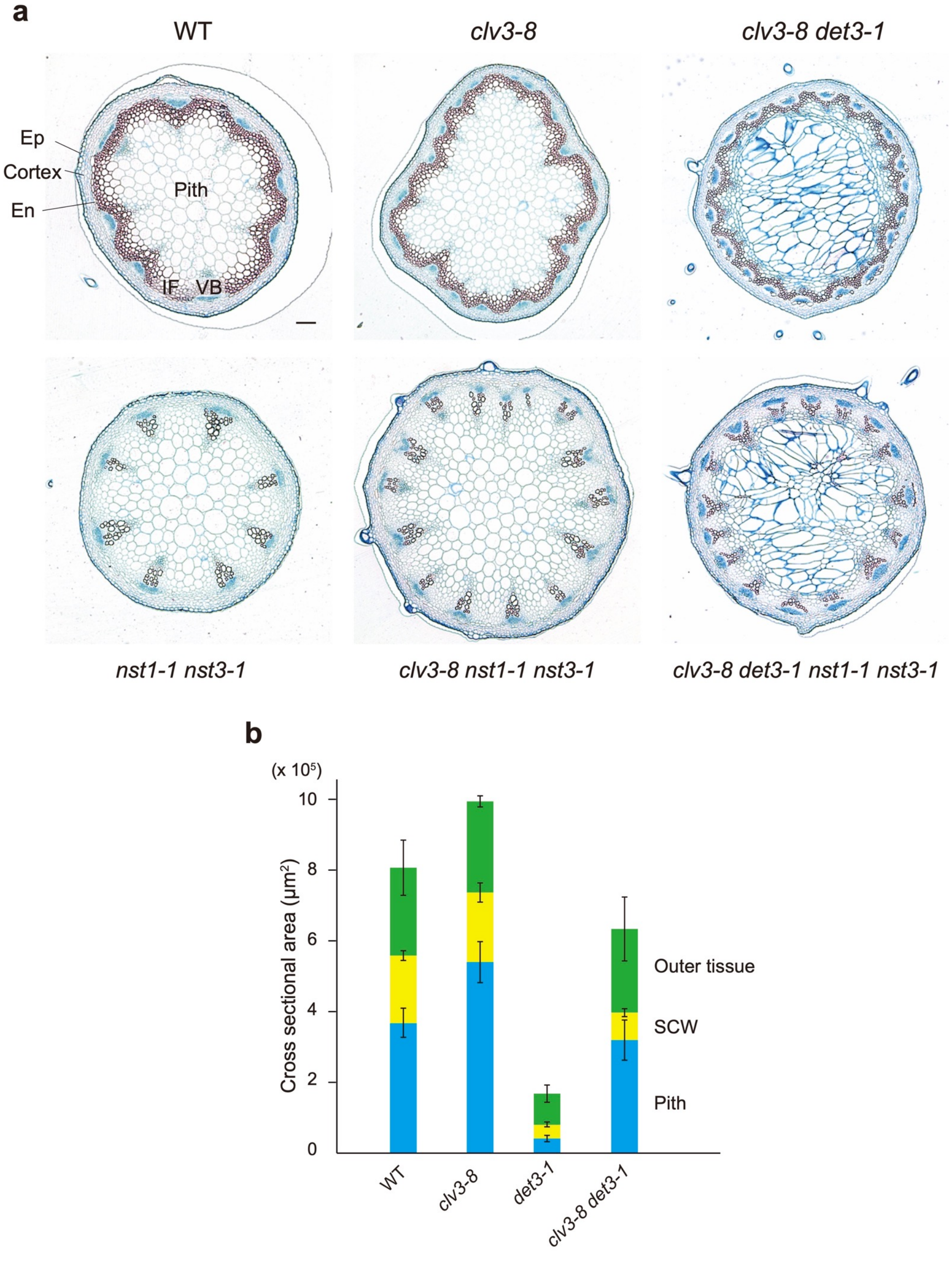


**Fig. S1**

Histological cross-sections showing inner tissue and quantification of area of stem tissues. (a) Representative images of stem cross-sections. Scale bar = 100 µm. Lignified cell walls were stained in red. Ep, epidermis; En, endodermis; IF, interfascicular fiber; VB, vascular bundle. Histological cross-sectioning was performed as described previously (Asaoka et al., 2021). (b) Average cross-sectional area of the stem (N = 8–12). Data were calculated from the cross-sections prepared from the 1st node of the main stem at 40 days after seed sowing. (a, b) Plants were grown at the same condition in Asaoka et al. (2023): Seeds were sown on rockwool, watered daily with 0.5 g $L^{-1}$ Hyponex solution (Hyponex, Tokyo, Japan), and grown in a growth room with a 16-h/8-h light/dark cycle and white light fluorescent lamps at 50 µmol $m^{-2}$ $s^{-1}$.

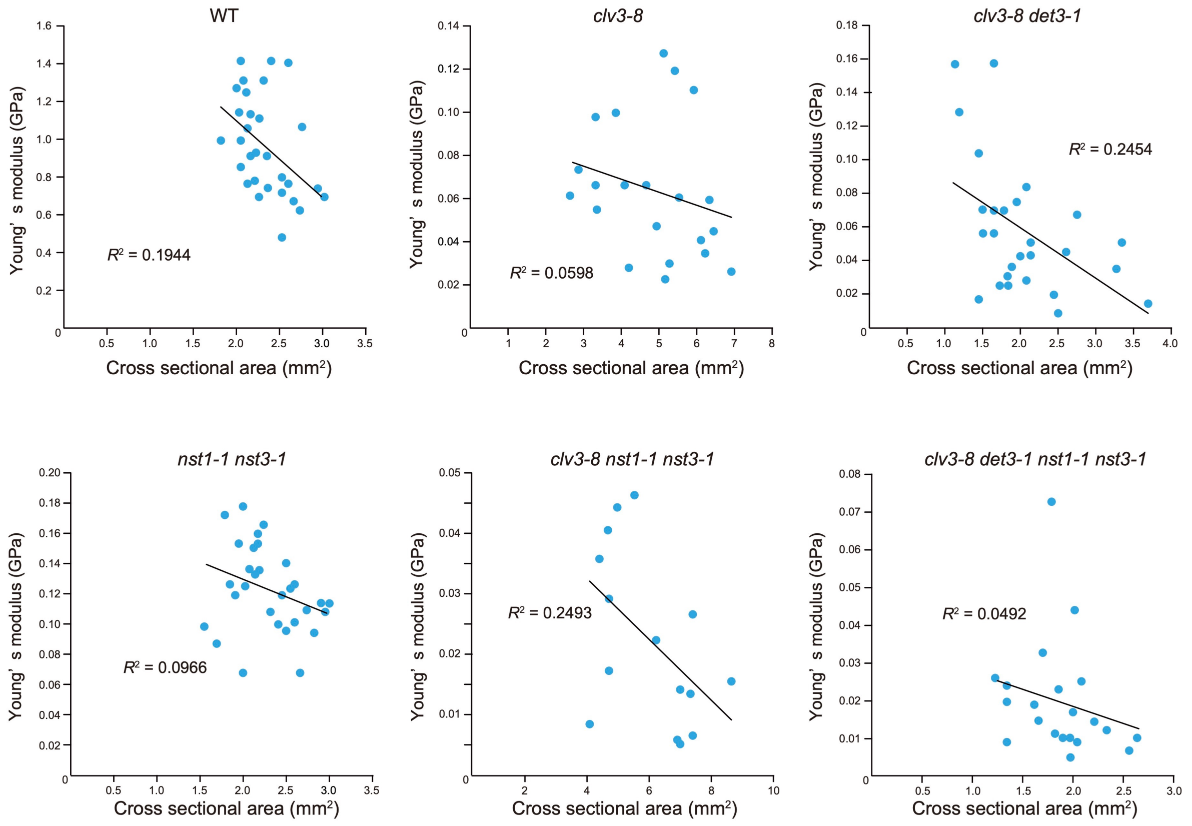


**Fig. S2**

Result of the three-point bending test based on the size of stem cross-sections. Correlations between stem cross-sectional area and Young's modulus were calculated.
